# Case report: Human seminal plasma allergy diagnosis for a woman with unexplained infertility

**DOI:** 10.3389/fmed.2024.1403477

**Published:** 2024-08-29

**Authors:** Gabija Didziokaite, Aida Kuznecovaite, Gabija Biliute, Violeta Kvedariene

**Affiliations:** ^1^Faculty of Medicine, Vilnius University, Vilnius, Lithuania; ^2^Department of Pathology, Institute of Biomedical Sciences, Faculty of Medicine, Vilnius University, Vilnius, Lithuania; ^3^Innovative Allergology Center, Vilnius, Lithuania

**Keywords:** human seminal plasma allergy, sperm allergy, HSP allergy, infertility, unexplained infertility, Can f 5, cross-reactivity, PSA

## Abstract

**Background:**

Infertility is a pressing global public health concern, affecting millions worldwide, and the diagnosis of unexplained infertility poses particular challenges. Human seminal plasma allergy, a rarely diagnosed type I hypersensitivity reaction, emerges as a potential but often overlooked contributor to female infertility. With rare reported cases globally, the condition’s low awareness and insufficient differential diagnosis may mask its actual prevalence.

**Case report:**

This case report presents the clinical case of a 29-year-old woman with unexplained infertility who underwent two unsuccessful IVF procedures and was subsequently diagnosed with human seminal plasma allergy. The patient, known for bronchial asthma and allergic rhinitis exacerbated by inhalant allergens, exhibited eosinophilia and a history of local allergy symptoms (burning sensation, vulvar pruritus, edema, and general discomfort) as well as sneezing and nasal congestion following unprotected intercourse—symptoms compatible with human seminal plasma allergy. Molecular allergy diagnostics revealed pronounced sensitization to dust mites and Can f 5, a canine-specific allergen. A positive skin prick test using her partner’s sperm confirmed the diagnosis of human seminal plasma allergy. The patient’s medical history also includes mild endometriosis, raising questions about the interplay between allergic conditions and fertility. Treatment options such as barrier contraception, antihistamine therapy, and sperm desensitization are discussed.

**Conclusion:**

Highlighting the need for increased awareness among healthcare professionals, this case emphasizes the significance of reporting and sharing clinical experiences to enhance our understanding of this rare condition. As researchers continue to accumulate relevant information, a more comprehensive understanding of human seminal plasma allergy and its potential impact on female fertility will contribute to improved diagnostic protocols and expanded treatment options. This case report contributes to the growing body of knowledge surrounding this rare allergy, serving as a reminder of possible intricate relationships between allergic conditions and reproductive health.

## 1 Introduction

Infertility is a major problem globally and a public health priority nowadays (1). It is defined as an inability to conceive within 1 year of unprotected intercourse (1). In 2002, the World Health Organization estimated that approximately 80 million individuals worldwide were impacted by infertility (2). This condition is known to affect approximately 17.5% of the adult population at some point in their lives (3) and as many as 20–50% of these cases are diagnosed as “infertility of unknown origin” or “unexplained infertility” (4–7). However, it’s important to note that accurately assessing the true prevalence of infertility can be challenging and is not accurate. Moreover, the diagnosis of unexplained infertility (UI) does not allow the couple to take targeted action to conceive and its treatment remains largely empirical. Therefore it is important to investigate other possible underlying causes of infertility, including rare allergies and other disorders of impaired immune system that may affect the reproductive system (8–10).

## 2 Case report

A 29-year-old woman with a history of bronchial asthma (BA) and allergic rhinitis (AR) was referred to the allergologist-immunologist for allergy testing. Her symptoms exacerbated by cat fur, dust and mold. There was no family history of allergic diseases. The patient was not able to conceive for 4 years. The thorough gynecological investigation has been carried out. No anatomical pathologies, infections or other possible causes of infertility were found. Mild endometriosis had been diagnosed, however, due to its low severity it was not considered to be the cause of infertility for this patient. Therefore, the diagnosis UI was established and the patient had already undergone 2 unsuccessful *in vitro* fertilization (IVF) procedures.

Upon admission, the patient exhibited eosinophilia, a finding consistent with the eosinophil count observed in blood samples over the past 4 years. In the most recent Complete Blood Count (CBC), the eosinophil count was 0.4 × 10^9^/L, accounting for 9.8% (normal range: 0–5%) of the total white blood cell count.

In assessing respiratory function, spirometry indicated well-controlled asthma. The forced expiratory volume in 1 s (FEV1) measured 3.56 L, representing 107% of the predicted value. Post-bronchodilator administration resulted in an 11% improvement, with the FEV1 increasing to 3.96 L. The forced vital capacity (FVC) was 4.31 L, surpassing the predicted value at 114%, and the FEV1/FVC ratio stood at 92%, aligning with the predicted ratio of 100%.

Additionally, nasal secretion cytology revealed eosinophilic leukocytes, constituting 29% of the current sample (normal range: 0–5%). This corroborates the systemic eosinophilia observed in blood samples and emphasizes the involvement of eosinophils in both the respiratory and nasal compartments.

Skin prick tests revealed pronounced sensitization to house dust mites, grass and weed pollen. ALEX2 (Allergy Explorer) macroarray test revealed pronounced sensitization to grass pollen, house dust mites, storage mites, dog, cat and insects’ allergens (Supplementary Table S1). Of particular note, the most pronounced sensitization was observed for Can f 5 (*Canis familiaris allergen 5*), a prostatic kallikrein found in dog’s urine: 26.58 kU/L. The latter finding was of particular importance to the allergologist-immunologist as the possible cross-reactivity with human seminal plasma (HSP) allergens was then suspected. Due to the possibility of HSP allergy, medical team conducted an interview with the patient regarding symptoms experienced after sexual intercourse without using a condom. The patient noted that sneezing and nasal congestion usually occurred after unprotected intercourse. To confirm the diagnosis of HSP allergy, a skin prick test was performed for the women on the following appointment, using seminal plasma extracted from the husband’s sperm. Preparation of the human seminal plasma for the skin prick involved incubating the woman’s partner’s sperm at room temperature for 30 min, followed by centrifugation at 1,000 rpm for 30 min to separate spermatozoa from seminal plasma proteins (Supplementary Figure S1). Following a waiting period of 15 min. Post-prick application, the resulting wheals were compared with the standard skin pricks: positive control with histamine and negative control solution. Wheals on the skin prick with seminal plasma indicated a positive reaction to sperm for the women with the wheal equal to histamine (as 3+ according to allergological evaluation). Her husband served as control (Supplementary Picture S1). Also, house dust mite sublingual immunotherapy was initiated, however, the patient stopped it soon.

In the follow-up visit after 3 years, the patient remained unable to conceive. She noted that some localized symptoms, characteristic to HSP allergy, has recently emerged: burning sensation, vulvar pruritus, edema and general discomfort occurred after unprotected sexual intercourse. Moreover, additional systemic allergy symptoms occurred after contacting semen, such as eye watering and puffy eyelids. According to the patient, mentioned local and systemic symptoms occurred after contacting her partner’s semen in about 50 percent of the cases. The ALEX2 macroarray test was performed repeatedly for the patient to exclude new sensitizations and similar results were obtained as 3 years ago (Supplementary Table S1). However, some additional sensitizations were detected and some previous sensitizations were decreased. ALEX2 results indicated pronounced sensitization to: grass pollen, house dust mites, storage mites, oysters, cats, dogs, mouse, rat and horse allergens, showing molecular spreading. Sensitization to Can f 5 remained the most pronounced: 45.06 kU/L, potentially explaining the worsening symptoms upon sperm exposure (Supplementary Table S2).

## 3 Discussion

Infertility poses a significant global health concern, defined as an inability to conceive within 1 year of unprotected intercourse (1). If the cause of infertility remains unidentified despite a thorough examination of both partners, it is defined as infertility of unknown origin or UI, affecting as many as 20–50% of couples seeking infertility care (5, 6). However, this diagnosis does not allow the couple to take targeted action to conceive and its treatment remains largely empirical. Therefore, the links to other diseases that could affect women’s fertility are currently being investigated as well as other underlying causes of infertility, including allergies and other disorders of impaired immune system (8–10).

Pronounced sensitivity to male sperm is a potential cause of female infertility (11, 12). Although male’s semen is composed of many components, the two most described hypersensitivity reactions are type I reactions to HSP and type II-like reactions to spermatozoa. Type I hypersensitivity reactions are called allergies and are detected to the usual components of the human sperm’s liquid part, most commonly to the prostate-specific antigen (PSA) (13). Also, there are some allergies described to certain other substances, which may get into seminal plasma after being consumed by a man, such as nuts or different medications (14–17). Although type II-like hypersensitivity reactions to spermatozoa are quite well known to the reproductive specialists and specific IgG and IgM class antibodies to the surface of spermatozoa may be detected as a part of routine examination for infertile couples, awareness of HSP allergy is still lacking among obstetricians-gynecologists, therefore, due to insufficient differential diagnosis, this allergy is diagnosed relatively rarely (11, 12, 18). In our presented case, the patient was also tested for anti-sperm antibodies by a reproductive specialist and none were found. However, neither her obstetrician-gynecologist, nor her reproductive specialist referred the patient to be investigated by immunologist-allergologist for further investigation due to her multiple allergies and specific symptoms following unprotected intercourse. Our patient was consulted by allergist for allergy investigation and possible type I hypersensitivity reaction to HSP was diagnosed only after careful allergy investigation.

To this day, human seminal plasma allergy is considered as an exceptionally rare phenomenon with fewer than 100 documented cases so far. HSP allergy predominantly affects women aged 20–30. The exact prevalence is unknown due to limited awareness and testing globally (19). This type of hypersensitivity reaction may affect women’s ability to conceive not only by causing distressing symptoms during unprotected intercourse but also by triggering allergic inflammation in the reproductive tract. This inflammation involves various immune cells like mast cells, lymphocytes, basophil eosinophils and sometimes neutrophils, along with the production of inflammatory mediators such as lipids, purines, cytokines, chemokines and reactive oxygen species (ROS) in type I hypersensitivity reactions (20, 21). Allergic inflammation may affect target cells such as epithelial cells, fibroblasts, vascular cells, and smooth muscle cells, causing them to become significant producers of inflammatory mediators. The coordination of allergic inflammatory responses engages a range of transcription factors, particularly NF-κB and GATA3 (20). As allergic reactions to HSP may occur not only locally, but also systemically, similar processes may also have a negative effect on other organs related to woman’s fertility, contributing to the creation of an adverse environment that may negatively affect woman’s fertility. In the case presented, the patient fits the typical demographic profile of seminal plasma allergy cases, being 20–30 years old. Moreover, she was also diagnosed with infertility and despite thorough gynecological investigation no causative agent of her infertility had been found yet. Therefore, allergic inflammation induced by HSP allergy, could be a plausible underlying cause for this patient’s UI.

The diagnosis of HSP allergy is established through a combination of medical history, the presence of specific IgE antibodies in the serum, and skin prick tests (22). The clinician should determine whether the woman has any signs of an immediate systemic or localized reaction following the exposure to HSP during unprotected sexual intercourse. The localized symptoms characteristic to HSP allergy are vaginal or vulvar pain, pruritus, burning sensation and/or edema, while systemic symptoms may include generalized urticaria, angioedema, diffuse pruritus, dyspnea, wheezing, gastrointestinal symptoms, and/or loss of consciousness (13). Anaphylactic reactions with vascular collapse caused by exposure to HSP during sexual intercourse are uncommon. Since the first documented case in 1958, there have been approximately 30 cases of anaphylaxis induced by HSP (13). The primary criterion for this diagnosis is the absence of symptoms when barrier contraception is used to prevent woman’s contact with semen during the intercourse (23). A gynecological evaluation is necessary to rule out other possible causes of vaginal discomfort, including organic pathologies and anatomical abnormalities, sexually transmitted diseases or chronic vaginal candidiasis (13). If HSP allergy is suspected for a patient, the gold standard to confirm this diagnosis is a skin-prick test with patient’s partner’s fresh seminal plasma, prepared by centrifuging the sperm sample for 30 min to separate the fluid from the spermatozoa. Also, serologic testing for IgE specific to HSP proteins is indicated as a possible alternative for diagnosis of HSP allergy, however, commercially available assays for this testing are not widely validated (13, 18). In our case, the patient lacked localized symptoms at first, but experienced sneezing and nasal congestion after intercourse, initially overlooked by specialists.

More than 50 years ago Halpern et al. proposed that allergy symptoms experienced by some women after their first unprotected sexual intercourse might be caused by a previous sensitization to unknown antigens that cross-react with HSP proteins (24). Cross-reactivity between HSP and dog allergen, specifically human prostate-specific antigen (PSA), was demonstrated 16 years ago by Basagaña et al. (25). In a research by Basagaña et al. published in 2012, dog dander allergen, that showed cross-reactivity with human PSA, has been identified as an IgE-binding protein prostatic kallikrein Can f 5, which is an androgen-regulated protein expressed in the prostate, and hence present only in male dogs (26, 27). Can f 5 has been first isolated from dog urine and identified by Mattsson et al. (28). It is a major dog allergen associated with respiratory allergies (AR, BA) (29). In our case, testing for HSP allergy was initiated only after molecular allergy diagnostics revealed sensitization to Can f 5. This finding, along with knowledge of possible cross-reactivity, led to a thorough patient interview and skin prick testing using the partner’s seminal plasma as the diagnostic gold standard for this condition.

Several options exist to prevent patients from experiencing the symptoms of HSP allergy. Using barrier contraception is the simplest recommendation, but it’s not suitable for couples trying to conceive. Another option, more suitable for couples in order to conceive, is prophylactic antihistamine therapy, suggested to be taken 1 h before each sexual intercourse to reduce the impact of allergic reactions and intensity of discomfort-inducing local allergy symptoms, however, success rates are limited, with only one reported case of successful conception (30).

The only known etiological treatment for HSP allergy is sperm desensitization, which involves gradually introducing increasing concentrations of semen into the woman’s body to develop tolerance (13, 31). There are two methods: intravaginal and subcutaneous (13). However, this treatment approach is not applied in every healthcare facility worldwide. In our case, as the patient was trying to conceive and desensitization was unavailable in our country, prophylactic antihistamine therapy was recommended, which she found ineffective.

For the couples with UI, options like IVF and intrauterine insemination (IUI) are available (32, 33). IUI may be preferable for UI without sperm hypersensitivity (34). Unfortunately, there is limited information and recommendations regarding treatment options for women who have HSP allergy. As allergies may trigger an inflammatory reaction in the body, certain methods are suggested to be applied in order to avoid the development of allergic reactions in women’s body and to conceive through IVF of IUI (13, 23). Methods like sperm washing before assisted reproductive technology (ART) procedures are suggested to prevent allergic reactions caused by traces of seminal plasma on the surface of spermatozoa (35). Iwahashi et al. in 1999 reported a successful artificial insemination using washed spermatozoa for a woman with sperm allergy, but it took six attempts (36). In 2010, Frapsauce et al. also documented successful insemination with washed spermatozoa despite continued allergic reactions. Antihistamine therapy was supplemented, and though the woman developed systemic erythema, fertilization occurred (35). In our case, despite undergoing two unsuccessful IVF cycles, sperm washing wasn’t offered due to non-standard practice.

Despite numerous reported cases of HSP allergy associated with infertility in some cases, including ours, there is still no conclusive evidence that it directly causes female infertility (32, 37, 38). In 2023 Tan et al. reported varying fertility outcomes for women experiencing localized HSP reactions. Out of 12 women who underwent subcutaneous desensitization, 7 had successful desensitization, among them, 3 successfully conceived and gave birth, 2 experienced miscarriages, 1 could not conceive and 1 did not attempt pregnancy. For 2 women, desensitization was partially successful, as their symptoms subsided. Both women later successfully conceived and delivered babies. Among the 3 women with unsuccessful desensitization, one still became pregnant. This suggests that successful desensitization is not a prerequisite for pregnancy, emphasizing the need for further evaluation of infertility causes beyond HSP allergy treatment (39). In our clinical case, apart from HSP allergy, other factors could have contributed to this patient’s infertility. Firstly, the patient had BA which previously was not treated appropriately. Current clinical trials show a clear trend linking female asthma and reduced fertility. Asthma is linked to prolonged time to pregnancy, lower pregnancy rates, increased miscarriages, and menstrual (8, 40–44). Treatment of asthma is crucial as it may reduce systemic inflammation and improve infertility outcomes, possibly reducing the need for *in vitro* fertilization (45). While the direct mechanism linking asthma to UI remains unclear, ongoing research aims to uncover this connection.

Our patient was also diagnosed with low-grade endometriosis. However, mild endometriosis itself is not considered to cause infertility (46, 47). Moreover, endometriosis is more prevalent in patients with asthma (48). In 2021 Shafrir et al. found that women with surgically-diagnosed endometriosis were 76% more likely to have allergies compared to those without endometriosis. Moreover, the odds of having endometriosis were statistically significantly higher for each allergy type analyzed separately, except for animal allergies (49). Our patient, who had mild endometriosis, was also diagnosed with several allergies, to cat, dog, house dust mites and mold, since her childhood.

In 2017, Peng et al. published a research claiming that the risk of endometriosis in asthma group was 1.50 times higher (with a 95% confidence interval ranging from 1.33 to 1.70) compared to the non-asthma group, particularly among patients aged 21–50 years (50). A reverse correlation has also been identified in recent studies. It has been revealed that endometriosis, a prevalent factor contributing to infertility, frequently co-occurs with allergic conditions. During the last 20 years of research, it was established that women diagnosed with endometriosis are more likely to experience AR, atopic dermatitis, allergic conjunctivitis, eczema and asthma compared to women without fertility issues. This intriguing connection underscores the complex interplay between reproductive health and allergic conditions (43, 51–54). This connection suggests a complex interplay between reproductive health and allergies, possibly linked to a Th1/Th2 immune response associated with endometriosis (48). Although endometriosis may not solely cause of infertility in our case, but it is an important finding highlighting ongoing inflammatory processes throughout the body.

A new hypothesis suggests that endometriosis may not only cause oxidative stress by catalyzing Fenton reaction but could be caused by oxidative stress itself and the finding of endometriosis could be a sign of elevated levels of reactive oxygen species in the body (55). Oxidative stress is also implicated in allergic diseases with studies highlighting biomarkers like nitrotyrosine (Tyr-NO2) and nuclear factor erythroid 2-related factor 2 (Nrf2) in mechanisms of asthma research (56, 57). Also, in 2021 oxidative stress and antioxidant pathways in AR were investigated by Han et al. (58). In cases where endometriosis and allergies coexist, oxidative stress levels may be particularly high, potentially affecting fertility by impairing follicular, hydrosalpingeal and peritoneal fluid microenvironments and impacting oocyte quality, implantation, and early embryonic development (59).

## 4 Conclusion

In conclusion, this case report sheds light on the often-overlooked association between human seminal plasma allergy and unexplained female infertility. With less than 100 documented cases globally, the condition’s low awareness poses challenges in diagnosis. This case serves as a reminder that seemingly unrelated allergic conditions, when combined, can contribute to reproductive health challenges, warranting comprehensive evaluations.

The patient’s history of BA and AR, if untreated, may have prolonged the time to pregnancy, aligning with current clinical trials linking female asthma to reduced fertility. The coexistence of low-grade endometriosis and sensitization to various allergens adds complexity to the inflammation, potentially impacting oocyte quality and early embryonic development. The identification of oxidative stress as a common element in endometriosis and allergic reactions further complicates the fertility landscape.

In navigating such cases, it becomes crucial for healthcare professionals to consider a holistic approach, addressing not only the primary diagnosis of human seminal plasma allergy but also the interconnected factors influencing reproductive health. Practical insights/treatment options such as barrier contraception, antihistamine therapy, and sperm desensitization should be used for managing this complex seminal plasma allergy.

As research progresses, a more comprehensive understanding of these intricate relationships will contribute to improved diagnostic protocols and tailored treatment options for individuals facing the complexities of unexplained infertility.

## Data Availability

The original contributions presented in the study are included in the article/Supplementary material, further inquiries can be directed to the corresponding author.
