# Supplementary material for: Case report: Human seminal plasma allergy diagnosis for a woman with unexplained infertility
Source: Front Med (Lausanne). 2024 Aug 29;11:1403477. doi: 10.3389/fmed.2024.1403477 (PMC11390391; doi:10.3389/fmed.2024.1403477)
Supplement: Supplementary file 1 [file Table_1.DOCX]

Supplementary Material

# Supplementary Data

# Supplementary Figures and Tables

## Supplementary Figures


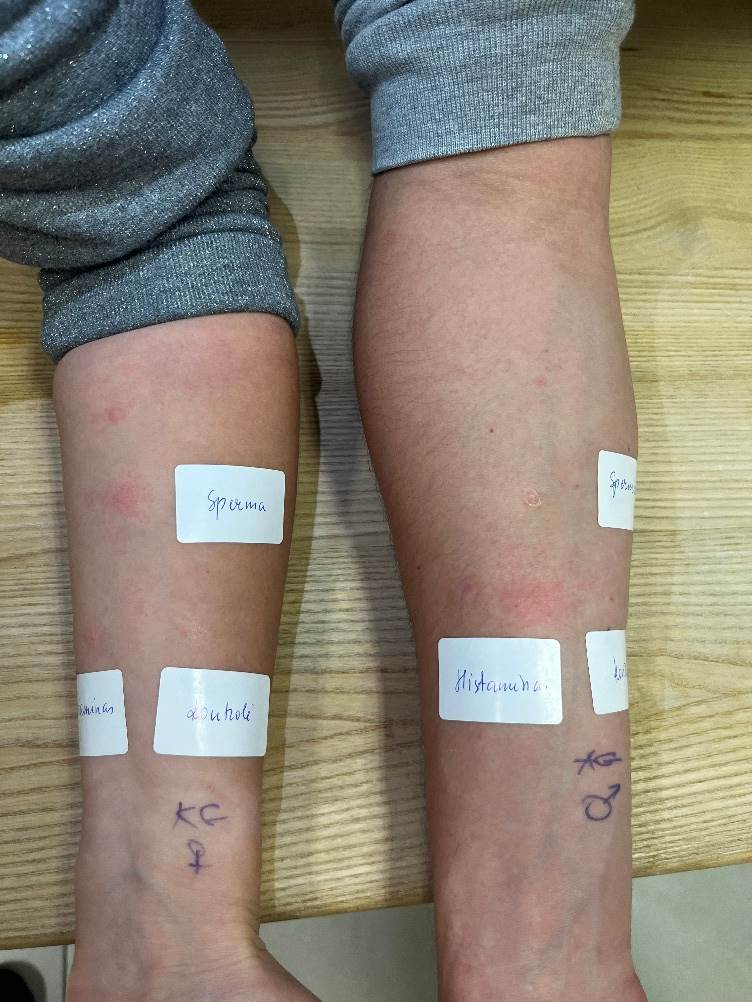


**Picture 1.** Results of the skin prick test. The picture shows wheels in both positive controls (histamine) and a positive reaction to human seminal plasma (the upper prick) on the woman’s arm (on the left). The prick with saline solution (negative control) did not react on anyone. No sensitization has been noticed on the woman’s partner’s arm (on the right). Human seminal plasma allergy was diagnosed for the woman. Images and clinical history were published with permission of the patient and her partner.


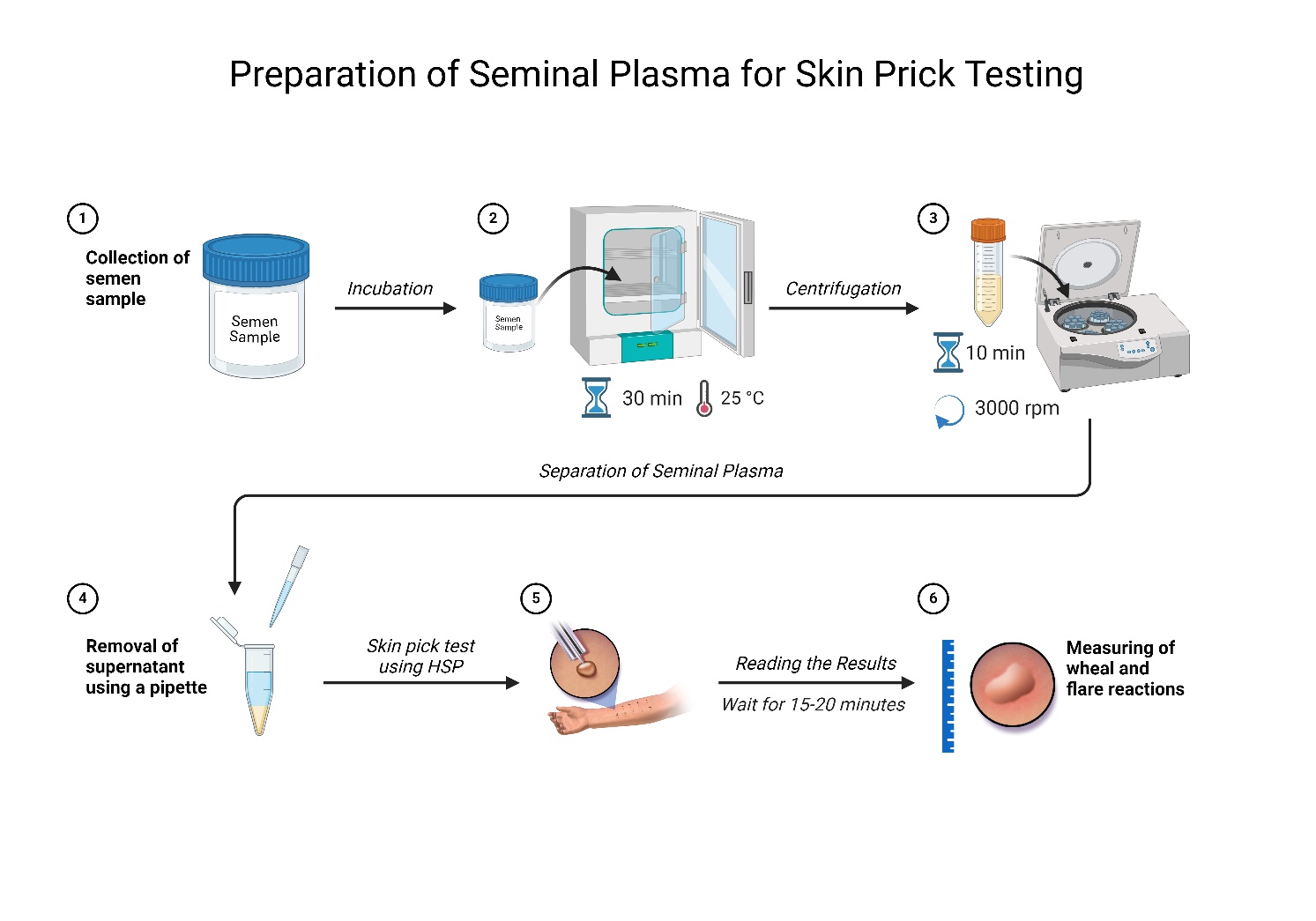


**Figure 1**: Preparation of Seminal Plasma and Skin Prick Test Procedure.
**1. Collection of Semen Sample:** The initial step involves the collection of a semen sample from the male partner. The sample is collected in a sterile container to ensure that it remains uncontaminated.
**2. Incubation:** The semen sample is allowed to incubate at room temperature for 30 minutes. This incubation period is necessary to liquefy the semen, making it easier to handle in subsequent steps.
**3. Centrifugation:** After incubation, the liquefied semen sample is carefully transferred into a centrifuge tube. This step prepares the sample for centrifugation, which will separate the seminal plasma from the sperm cells. The sample in the centrifuge tube is subjected to centrifugation at 3000 rpm for 10 minutes. Centrifugation separates the seminal plasma (supernatant) from the sperm cells (pellet).
**4. Separation of Seminal Plasma:** Post-centrifugation, the seminal plasma is carefully removed using a pipette. This supernatant contains the proteins and other components that may cause an allergic reaction.
**5. Skin prick test with HSP:** The skin prick test begins with preparing the test area, typically on the forearm or back. The skin is cleaned with alcohol to remove any surface contaminants. Drops of the prepared seminal plasma and control solutions (positive control like histamine and negative control like saline) are applied to the skin. Using a sterile lancet, the skin is pricked through each drop of the applied solutions. This allows the allergen to penetrate the skin and elicit a reaction if there is a sensitivity.
6**. Reading the Results:** The test area is left undisturbed for about 15-20 minutes to allow for a reaction to develop. During this time, any wheal (raised bump) and flare (redness around the wheal) will become visible. The results of the skin prick test are evaluated by measuring the size of the wheal and flare reactions. A positive reaction indicates an allergy to the seminal plasma.

## Supplementary Tables

**Table 1.** Patient’s sensitization according to ALEX2 macroarray test: on admission and follow-up after 3 years.

**Table 2.** A timeline with relevant data from the episode of care described.
